# Shared Pathogenic Features Between Serotonin Receptor Antagonist-Associated Diabetic Ketosis and Ketosis-Prone Type 2 Diabetes: A Case Report

**DOI:** 10.7759/cureus.82833

**Published:** 2025-04-23

**Authors:** Shumpei Nakanishi, Haruhiko Sato, Yoichi Oikawa, Kaori Ikebukuro, Akira Shimada

**Affiliations:** 1 Department of Endocrinology and Diabetes, Saitama Medical University, Saitama, JPN; 2 Department of Internal Medicine, Tsurugashima Medical Clinic, Saitama, JPN

**Keywords:** 5-hydroxyindoleacetic acid, diabetic ketosis/ketoacidosis, ketosis-prone type 2 diabetes, serotonin, serotonin receptor antagonist

## Abstract

Ketosis-prone type 2 diabetes (KPD) is characterized by male predominance, onset at a young age, obesity, and sudden onset of diabetic ketosis/ketoacidosis without precipitating factors, negative anti-islet autoantibodies, and β-cell function preservation after recovery from diabetic ketosis/ketoacidosis following temporal insulin therapy. However, its pathogenesis remains unknown. We encountered a 49-year-old obese man presenting with diabetic ketosis, i.e., ketonuria, plasma glucose 252 mg/dL, HbA1c 12.8%, without anti-islet autoantibodies, induced by dose escalation of quetiapine, a serotonin receptor antagonist. After discontinuing quetiapine and starting subcutaneous intensive insulin therapy, diabetic ketosis rapidly resolved. Following glycemic state stabilization, insulin therapy was discontinued on the 11^th^ day of the initiation of therapy. Instead, metformin and linagliptin were initiated, and his glycemic status remained well controlled thereafter. His clinical course closely resembled that of KPD, together with our literature review, suggesting the involvement of decreased serotonin production/action in the pathogenesis of KPD.

## Introduction

Diabetes mellitus is a metabolic disease that is characterized by chronic hyperglycemia, including two major types of disease, that is, type 1 diabetes (T1D) and type 2 diabetes (T2D). In T1D, absolute insulin deficiency is associated with autoimmune destruction of the pancreatic β-cells. People with typical T1D usually experience diabetic ketosis/ketoacidosis (DK/DKA) due to absolute insulin deficiency from the onset of the disease and therefore require insulin therapy very early in the clinical course. On the other hand, T2D is a heterogeneous metabolic disorder characterized by insulin resistance along with varying degrees of insulin secretory defects. Because insulin secretion is generally preserved to some extent in people with T2D, DK/DKA does not generally occur unless triggered by a severe infection, etc. However, in recent years, attention has been drawn to the existence of a subtype of T2D in which DK/DKA easily develops and/or repeats without any precipitating factors, that is, ketosis-prone type 2 diabetes (KPD) [1].

KPD is characterized by several distinctive features. Concretely, it predominantly affects young men with obesity and is marked by recurrent episodes of DK/DKA without other contributing factors, accompanied by sudden weight loss following the attainment of maximal body weight (BW) [1]. Upon initiation of intensive insulin therapy, individuals with KPD typically experience a restoration of normal glucose tolerance [2]. However, the underlying cause of KPD in individuals with obesity remains unclear. We recently encountered a man with obesity who exhibited a KPD-like clinical presentation following dose escalation of quetiapine, a serotonin receptor antagonist. Furthermore, we investigated the clinical features of previously reported quetiapine-associated DK/DKA cases and found that many of the cases also showed clinical features like those of KPD. Therefore, this report aimed to summarize these clinical features and discuss the pathogenesis of KPD with a focus on serotonin signaling.

## Case presentation

**Figure 1:**
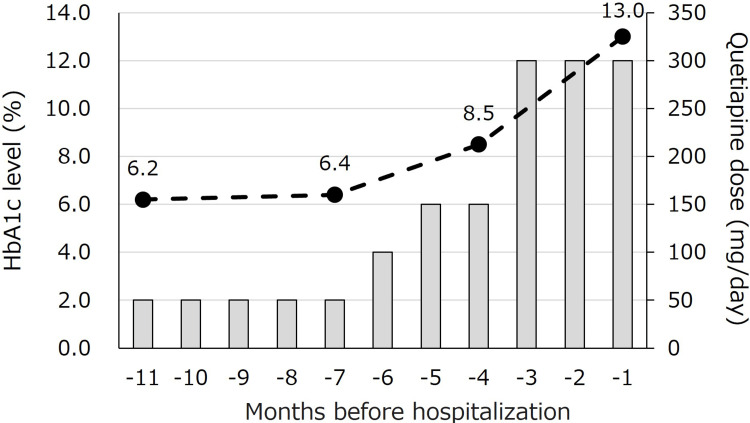
Time courses of quetiapine dose and HbA1c levels until hospitalization. Five months prior to admission, the quetiapine dose was increased from 50 to 100 mg/day, resulting in impaired glucose tolerance. When the dose was further increased to 300 mg/day, HbA1c levels increased to 13.0%. The dotted line and solid squares represent the HbA1c level and quetiapine dose, respectively.

A 49-year-old man, with a height of 165 cm, a BW of 89.4 kg, and a body mass index of 32.8 kg/m², had been under psychiatric care for bipolar disorder since the age of 30. Quetiapine therapy (50 mg/day) was initiated nine years before presentation. Since the patient had experienced worsening symptoms of depression, the quetiapine dose was doubled to 100 mg/day six months earlier, impairing glucose tolerance (Figure 1).

Upon increasing the dose to 300 mg/day, the patient experienced a BW loss of 11 kg (from 100 kg to 89 kg) within three months despite no change in dietary intake or physical activity, and the HbA1c level increased to 13.0%. Before weight loss began, the patient had maintained his highest weight of approximately 100 kg in recent years. Owing to increased thirst, his metabolic status was evaluated thereafter, revealing a DK state (plasma glucose 252 mg/dL, HbA1c 12.8%, and urinary ketone body 2+) (Table 1). He had no history of consuming large amounts of soft drinks, and there were no specific precipitating factors for weight loss.

**Table 1:** Main laboratory data of the patient on admission. On admission, the patient was in a state of diabetic ketosis, with a casual plasma glucose level of 252 mg/dL, a hemoglobin A1c level of 12.8%, and a urinary ketone body level of 2+. Anti-GAD antibody was negative. GAD: glutamic acid decarboxylase; TgAb: thyroglobulin antibody; TPOAb: thyroid peroxidase antibody; TRAb: thyroid-stimulating hormone receptor antibody; TSH: thyroid-stimulating hormone; eGFR: estimated glomerular filtration rate

Complete blood count	Normal range	Measurements
White blood cell (×1,000/μL)	3.30–8.60	12.26
Hemoglobin (g/dL)	13.7–16.8	16.3
Hematocrit (%)	40.7–50.1	46.8
Platelet (×1,000/μL)	158–348	185
Biochemistry	Normal range	
Albumin (g/dL)	3.9-4.9	4.6
Creatinine (mg/dL)	0.43–1.08	0.89
Blood urea nitrogen (mg/dL)	8.0–20.0	13.4
eGFR (mL/min/1.73 m^2^)	≥60.0	72.1
Aspartate aminotransferase (U/L)	10–37	84
Alanine transaminase (U/L)	5–40	87
Lactate dehydrogenase (U/L)	107–220	261
Alkaline phosphatase (U/L)	96–284	345
γ-Glutamyl transpeptidase (U/L)	0–73	131
Total bilirubin (mg/dL)	0.3–1.2	0.7
Amylase (U/L)	37–125	67
Casual plasma glucose	70–109	252
Hemoglobin A1c (%)	4.6–6.2	12.8
Anti-GAD antibody (U/mL)	<5.0	0.9
Free triiodothyronine (pg/mL)	1.71–3.71	2.91
Free thyroxine (ng/dL)	0.70–1.48	1.80
TSH (μIU/mL)	0.35–4.94	1.33
TRAb (IU/L)	≤2.0	<0.3
TgAb (IU/mL)	≤28.0	<10
TPOAb (IU/mL)	≤16.0	11
Urine test	(Normal range)	
Glucose (mg/dL)	(–)	500
Ketones body	(–)	(2+)
Protein (mg/dL)	(–)	30

**Figure 2:**
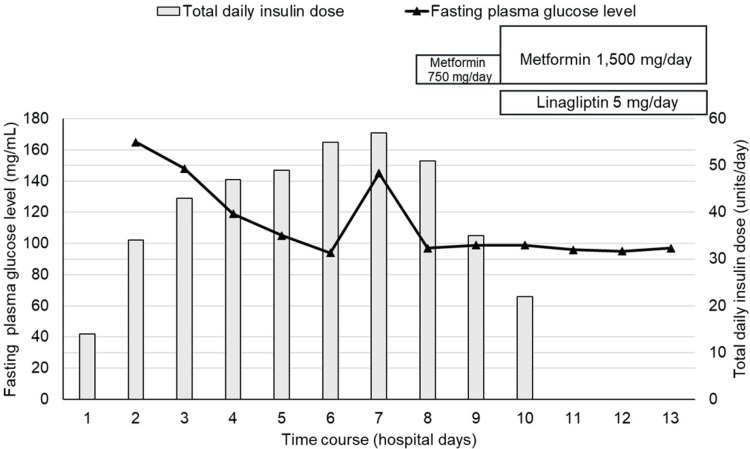
Time courses of fasting plasma glucose level and total daily insulin dose during hospitalization. Immediately after admission, subcutaneous intensive insulin therapy was initiated, and diabetic ketosis rapidly improved. Subsequently, because his glycemic state stabilized, insulin therapy was discontinued on the 11^th^ day of hospitalization. Instead, metformin and linagliptin were administered on days eight and 10 of hospitalization, respectively. Thereafter, his glycemic state remained well-controlled with these two oral hypoglycemic agents. The solid line and solid squares represent the fasting plasma glucose level and total daily insulin dose, respectively.

Given these findings, quetiapine was discontinued, and the patient was immediately admitted to our hospital for further treatment. The anti-glutamic acid decarboxylase antibody was negative. The fasting serum C-peptide level was 3.68 ng/mL (plasma glucose 178 mg/dL), the serum C-peptide level was 4.42 ng/mL two hours after a meal (plasma glucose 284 mg/dL), and the 24-hour urinary C-peptide value on the fourth day after admission was 265.03 μg/day, suggesting that an insulin secretory capacity was maintained to a certain extent. The patient had no microvascular or macrovascular diabetic complications at presentation and no family history of diabetes. As he maintained his appetite and was in good general condition, subcutaneous intensive insulin therapy was initiated without the need for intravenous insulin infusion, rapidly resolving the DK. Although the daily insulin dose requirement was temporarily increased to 57 units following glycemic state stabilization, insulin therapy was discontinued on the 11^th^ hospital day. Metformin was initially started at 750 mg/day on the eighth hospital day and increased to 1,500 mg/day in two days, and linagliptin (5 mg/day) was initiated on the 10^th^ hospital day (Figure 2). Nonetheless, his glycemic state remained well controlled with the two oral hypoglycemic agents. For reference, we selected linagliptin based not only on its efficacy but also on its safety, adherence, and ease of use; that is, it can be prescribed regardless of whether the patient has liver or renal impairment, it only needs to be taken once a day, and no dose adjustment is required.

## Discussion

In this case, the dose escalation of quetiapine may have led to DK development. However, after quetiapine discontinuation and intensive insulin therapy, the patient fully recovered to a stable glycemic state without needing maintenance insulin therapy. The clinical course is similar to that of KPD. DK/DKA caused by atypical antipsychotic agents, including quetiapine, is more prevalent in African American individuals and middle-aged men, and its clinical presentation resembles that of KPD [3]. Table 2 summarizes clinical features of 12 cases of DK or DKA associated with quetiapine previously reported in journals, for which at least clinical data regarding body mass index, duration of taking quetiapine, and its dose were available on PubMed using the following keywords: DK, DKA, and quetiapine. The mean (± standard deviation) age was 46.0 (± 14.8) years, and eight of the 12 cases were men. Nine and seven of the 12 cases had a BMI of >25 kg/m² and >30 kg/m², respectively. Despite presenting with DKA, seven of eight cases showed clinical courses that did not require insulin treatment after recovery from DKA, with discontinuation of quetiapine and insulin treatment. These clinical pictures, which are characterized by male predominance, onset at a relatively young age, obesity, onset of DK/DKA, and β-cell function preservation after recovery from DKA, have much in common with those of KPD.

**Table 2:** Cases of diabetic ketosis/ketoacidosis related to quetiapine administration. This table summarizes the clinical features of 12 cases of DK or DKA associated with quetiapine previously reported in PubMed journals using the following keywords: diabetic ketosis, diabetic ketoacidosis, and quetiapine. These clinical pictures, which are characterized by male predominance, onset at a relatively young age, obesity, onset of DK/DKA, and β-cell function preservation after recovery from DKA, have much in common with those of ketosis-prone type 2 diabetes. BMI: body mass index; DK: diabetic ketosis; DKA: diabetic ketoacidosis; HbA1c: glycohemoglobin; PG: plasma glucose; Ref: reference

No.	Age	Gender	Psychic disorders	Dose of quetiapine just before DK/DKA (mg/day)	BMI (kg/m^2^)	History of obesity	History of diabetes	Familial history of diabetes	Duration of taking quetiapine	PG level (mg/dL)	HbA1c (%)	Types of hyperglycemic crisis	pH	Final medication for diabetes after recovery from DK/DKA	Ref. No.
1	64	Man	Schizophrenia	400	30.2	Yes	ー	Yes	2 months	ー	ー	DKA	ー	ー	[4]
2	48	Man	Schizophrenia	800	19.1	No	No	ー	1 month	859	12.1	DKA	ー	Metformin	[5]
3	72	Man	Dementia	50	21.3	No	No	No	2 weeks	793	ー	DKA	7.294	None	[6]
4	51	Woman	Schizophrenia	400	30.7	Yes	No	No	24 months	954	7.2	DKA	7.1	None	[7]
5	33	Man	Schizophrenia	600	29	Yes	No	No	1 month	1422	ー	DKA	7.1	Insulin	[8]
6	45	Man	Schizophrenia	800	27.6	Yes	No	ー	5 months	1492	ー	DKA	7.148	None	[9]
7	41	Woman	Depression	400	37.2	Yes	No	No	1 month	1472	ー	DKA	7.022	ー	[10]
8	30	Woman	Schizophrenia	200	30-35	Yes	No	No	2 months	1081	ー	DKA	ー	ー	[11]
9	27	Man	Anxiety disorder	400	33.9	Yes	No	No	12 months	504	ー	DKA	7.35	(Death)	[12]
10	66	Man	Depression	25	22.8	Yes	No	Yes	6 months	1,709	13.1	DKA	7.213	None	[13]
11	40	Woman	Depression	400	40.9	Yes	No	No	10 months	443	9.2	DKA	7.1	Metformin	[14]
12	35	Man	Schizophrenia	400	39.6	Yes	No	ー	9 months	970	14.4	DKA	6.848	None	[15]
This case	49	Man	Bipolar disorder	300	32.8	Yes	No	No	9 years	252	12.8	DK	ー	Metformin and Linagliptin	ー

In this case, as the quetiapine pharmacological effects were exerted by inhibiting serotonin action, we hypothesized that a serotonin metabolism change may be involved in KPD pathogenesis. In this regard, we previously confirmed that the serum levels of serotonin and 5-hydroxyindoleacetic acid (5-HIAA), a final product of serotonin metabolism reflecting the serotonin amount in the whole body [16], were lower in patients with KPD than in those with T2D (unpublished data), suggesting the involvement of decreased serotonin production/action in the development of KPD.

The pancreatic β-cell mass increases in response to serotonin during pregnancy [17]. Selective serotonin reuptake inhibitors can enhance human β-cell mass in vitro, suggesting that elevated serotonin levels may promote β-cell proliferation [18]. Furthermore, serotonin signaling is associated with increased β-cell mass during puberty, a period characterized by insulin resistance [19]. Therefore, we hypothesized that an insulin-resistant state in individuals with obesity may be compensated by an increase in β-cell mass, but that a decrease in serotonin production or action may disrupt this compensatory mechanism, leading to sudden β-cell dysfunction followed by DK/DKA development in persons with KPD.

In this case, because serum levels of serotonin and 5-HIAA were not measured, it is unclear whether serotonin secretion recovered after the discontinuation of quetiapine. If these concentrations had been measured, the association between serotonin metabolism and the onset of DK might have been more strongly demonstrated, which is a limitation of this study. However, it is more likely that the restoration of serotonin action due to the discontinuation of quetiapine contributed to the recovery of insulin secretion capacity, along with the normalization of blood glucose levels through intensive insulin therapy. This requires further investigation.

Serotonin receptor antagonists are well known to worsen glucose tolerance. However, not all patients treated with serotonin receptor antagonists develop DK. We believe that the onset of DK cannot be fully explained by the action of serotonin alone. Various complex factors, such as the degree of serotonin action deficiency caused by serotonin receptor antagonists, BW, or changes in BW, and the reserve capacity of β-cell function, may be involved in the onset of DK. On the other hand, many drugs other than serotonin receptor antagonists can also worsen glucose tolerance, leading to DK. Thus, it is necessary to clarify the onset mechanism of DK caused by psychotropic drugs that have different effects than serotonin.

Generally, when mental states become unstable, various changes in dietary intake and physical activity may occur, possibly affecting lifestyle and glycemic control. However, in the present case, there was no change in dietary intake or physical activity, despite the patient's mental instability. Presumably, increasing the quetiapine dose minimizes appetite changes caused by worsening depression. Therefore, we believe that lifestyle changes due to mental instability are unlikely to have contributed to the onset of DK.

Unfortunately, we had no chance to measure blood total ketone body levels or plasma β-hydroxybutyrate levels and could not perform an accurate metabolic assessment based on these findings, which is one of the limitations of this case report. However, a urine test was performed by the patient's family doctor on the day before admission, and the result was urinary ketone 3+. Therefore, we believe that the patient was in a state of relatively strong DK before admission.

## Conclusions

We encountered a 49-year-old man with obesity who presented with DK induced by dose escalation of quetiapine. As his clinical presentation resembled that of KPD, together with our literature review, suggesting that decreased serotonin production or action may be involved in the development of KPD. In the future, it will be necessary to compare the serotonin metabolic status of serotonin receptor antagonist-associated DK/DKA cases with that of typical KPD cases and to clarify the involvement of serotonin signaling in the pathogenesis of KPD.
